# Supplementary material for: MetaQTL: a package of new computational methods for the meta-analysis of QTL mapping experiments
Source: BMC Bioinformatics. 2007 Feb 8;8:49. doi: 10.1186/1471-2105-8-49 (PMC1808479; doi:10.1186/1471-2105-8-49)
Supplement: Additional File 7 — MetaQTL Package : jar file and tutorial. This Zip archive contains both the MetaQTL JAR file and the files of the tutorial. [file 1471-2105-8-49-S7.zip › org.inra.metaqtl/doc/org/metaqtl/bio/entity/adapter/GeneticMapBeanAdapter.html]

GeneticMapBeanAdapter


|  |  |  |
| --- | --- | --- |
| **Overview** \| **Package** \| **Class** \| **Use** \| **Tree** \| **Deprecated** \| **Index** \| **Help** | |  |
| **PREV CLASS**   **NEXT CLASS** | **FRAMES**    **NO FRAMES**     **All Classes** |  |
| SUMMARY: NESTED \| FIELD \| CONSTR \| METHOD | DETAIL: FIELD \| CONSTR \| METHOD |  |


---


## org.metaqtl.bio.entity.adapter Class GeneticMapBeanAdapter

```
java.lang.Object
  org.metaqtl.bio.entity.adapter.BioEntityAdapter
      org.metaqtl.bio.entity.adapter.GeneticMapBeanAdapter
```

**All Implemented Interfaces:**: IBioAdapter

---

``` public class GeneticMapBeanAdapter extends BioEntityAdapter implements IBioAdapter ```

Class Description Here

**Author:**
:   Jean-Baptiste Veyrieras

---

**Constructor Summary**

`GeneticMapBeanAdapter()`


| **Method Summary** | |
| --- | --- |
| `java.lang.Object` | `fromEntity(IBioEntity obj)`             Convert the given compliant object to a bio entity object. |
| `IBioEntity` | `toEntity(java.lang.Object bean)`             Convert the given bio entity object into another compliant object. |

**Methods inherited from class org.metaqtl.bio.entity.adapter.BioEntityAdapter**

`fromEntity, toEntity`

**Methods inherited from class java.lang.Object**

`clone, equals, finalize, getClass, hashCode, notify, notifyAll, toString, wait, wait, wait`

**Constructor Detail**

### GeneticMapBeanAdapter

```
public GeneticMapBeanAdapter()
```


**Method Detail**

### toEntity

```
public IBioEntity toEntity(java.lang.Object bean)
```

:   **Description copied from interface: `IBioAdapter`**
:   Convert the given bio entity object into
    another compliant object.

    :   **Specified by:**: `toEntity` in interface `IBioAdapter`
    :   **Returns:**

---


### fromEntity

```
public java.lang.Object fromEntity(IBioEntity obj)
```

:   **Description copied from interface: `IBioAdapter`**
:   Convert the given compliant object to a
    bio entity object.

    :   **Specified by:**: `fromEntity` in interface `IBioAdapter`
    :   **Returns:**


---


|  |  |  |
| --- | --- | --- |
| **Overview** \| **Package** \| **Class** \| **Use** \| **Tree** \| **Deprecated** \| **Index** \| **Help** | |  |
| **PREV CLASS**   **NEXT CLASS** | **FRAMES**    **NO FRAMES**     **All Classes** |  |
| SUMMARY: NESTED \| FIELD \| CONSTR \| METHOD | DETAIL: FIELD \| CONSTR \| METHOD |  |


---
